# Comprehensive analysis of N6-methyladenosine -related long non-coding RNAs and immune cell infiltration in hepatocellular carcinoma

**DOI:** 10.1080/21655979.2021.1923381

**Published:** 2021-05-06

**Authors:** Zhong Lin Yu, Zheng Ming Zhu

**Affiliations:** Department of Gastrointestinal Surgery, The Second Affiliated Hospital of Nanchang University, Nanchang, China

**Keywords:** Bioinformatics analysis, n6-methyladenosine, lncRNAs, immune cell infiltration, hepatocellular carcinoma

## Abstract

We aimed to illustrate the influence of N6-methyladenosine (m6A) long non-coding RNAs (lncRNAs) and immune cell infiltration in hepatocellular carcinoma (HCC). The relationship of lncRNAs and m6A was identified through gene expression analysis using PERL and R packages. The Kyoto Encyclopedia of Genes and Genomes gene expression enrichment analysis was performed via gene set enrichment analysis. Lasso regression was utilized to construct prognostic model. Differences in the tumor microenvironment and the immune correlation were analyzed to clarify immune cell infiltration in different clusters and their correlation with the clinical prognosis. Co-expression analysis showed that lncRNA expression was associated closely with m6A. Many lncRNAs were predictive risk factors of prognosis in HCC. m6A-lncRNAs were partially highly expressed in tumor tissue and could be used in a prognostic model to predict HCC prognosis, independent of other clinical characteristics. ‘NOTCH SIGNALING PATHWAY’ was most significantly enriched according to GSEA. CKLF-like MARVEL transmembrane domain-containing member 3 (CMTM3) was overexpressed in tumor tissue. Immune cells, such as activated CD4 memory T cells, CD8 T cells, and follicular helper T cells, highly infiltrated tissues in cluster 2. All related scores were higher in cluster 2, indicating a lower purity of tumor cells and higher density of immune-related cells in the tumor microenvironment. m6A-lncRNAs are closely related to HCC occurrence and progression. Corresponding prognostic models can help predict HCC prognosis. m6A-lncRNAs and the related immune cell infiltration in the tumor microenvironment can provide novel therapeutic targets in HCC that need to be further studied.

**Figure:**
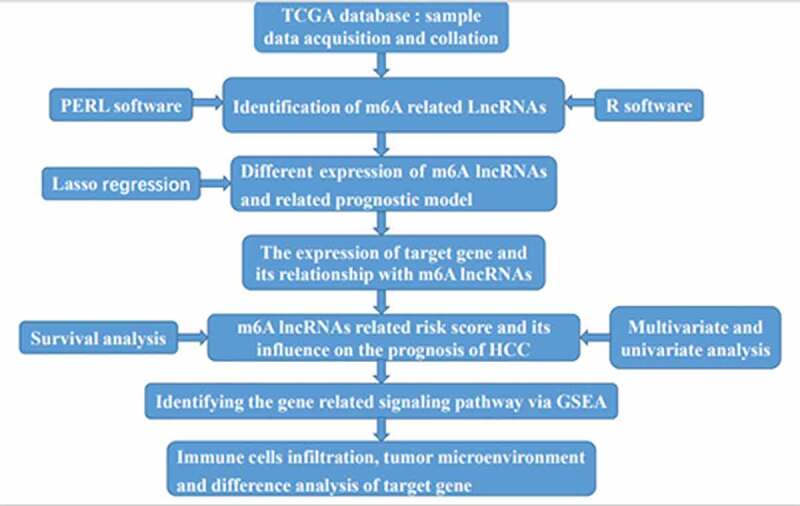
Graphical Abstract

Graphical Abstract

## INTRODUCTION

In 2020, the itinerary of the fight against cancer was hampered by the coronavirus disease (COVID-19) pandemic. Globally, China has the highest incidence and mortality rates of hepatocellular carcinoma for many years. Thus, effective treatments for advanced metastatic HCC are needed. One of the fundamental factors that accelerate the occurrence and progression of HCC is the accumulation of epigenetic alterations [1].

Aberrant N6-methyladenosine (m6A) modification stimulates tumor stem cell self-renewal, which further contributes to the progression of tumorigenesis, such as in HCC [2]. However, m6A can affect tumor development by mediating both tumor progression and tumor suppression; accordingly, a novel anticancer strategy involves the restoration of balanced RNA methylation in tumor cells [3,4]. There is increasing evidence that long non-coding RNAs (lncRNAs) are involved in many cancers because of their large number and expression specificity, and facilitating the elucidation of functional cancer-associated transcripts would be beneficial for the development of therapeutic interventions against cancer [5,6].

The m6A-methylated lncRNAs have an important regulatory effect on various biological and pathological processes. In osteosarcoma cells, ALKBH5-mediated mA modification of PVT1 contributes to osteosarcoma tumorigenesis [7]. METTL14 downregulation plays an essential role in the survival of HCC cells and stimulates the metastatic potential of HCC. Furthermore, METTL14 knockdown heightens the proliferative and invasive potential of HCC cells as well as promotes the tumorigenicity and metastasis via the modulation of m6A-dependent primary microRNA processing [2]. However, the deranged expression and m6A methylation of lncRNAs in HCC remains unelucidated. Therefore, the creation of a transcriptome map of the expression and m6A modification of lncRNAs in HCC has great significance in understanding the lncRNA-related mechanisms that affect the prognosis of HCC patients. Immune checkpoint-related gene signature has a potential clinical application in the risk estimation and survival prediction in HCC patients, and could be a potential indicator of the response to immunotherapy [8]. The diagnostic immune marker signature has been validated to accurately differentiate HCC from the adjacent tissues, and immune cell infiltration with upregulation of SLC41A3 expression in primary HCC is an independent prognostic factor for HCC patients [9]. However, there is limited research evidence of the correlation between m6A-lncRNAs and immune cell infiltration in HCC. Thus, it is imperative to analyze the immune cell infiltration in the tumor microenvironment (TME) to clarify their relationship with the clinicopathological parameters of HCC.

This study was conducted with an aim to identify m6A-related lncRNAs, whose expression is correlated closely with the prognosis of HCC patients, in a prognostic model that was developed to facilitate the prediction of HCC prognosis. Furthermore, the role of immune cell infiltration in the TME was identified. The elucidation of m6A-lncRNAs and the related immune cell infiltration in the TME was studied to help identify novel therapeutic targets and potential pharmacotherapeutic candidates for HCC treatment.

## MATERIALS AND METHODS

### Sample data acquisition and collation

We downloaded HCC gene expression profiles and clinical data from the Cancer Genome Atlas (TCGA) through the Genomic Data Commons Data Portal [10] (https://portal.gdc.cancer.gov/). In February 2021, the TCGA public database included the expression profiles of 407 HCC and 58 normal tissue specimens. We adopted the mRNA matrix via PERL software (https://www.perl.org/) and its corresponding script to organize transcriptome data and transform the IDs of the genes. The same software and specific script were used for the clinical data management in accordance with the developer’s instructions. Based on the guidelines released by the National Cancer Institute in December 2015 (https://cancergenome.nih.gov/publications/publication guidelines), our research did not require the approval of an ethics committee.

### Identification of m6A-related lncRNAs

Using PERL software, we constructed a gene expression matrix and human configuration file that contained information on the expression of m6A-related lncRNA gene profiles to distinguish between mRNA and lncRNA by using the collated transcriptome data. By running the corresponding script program, we derived the attributes of related genes and obtained the respective data of mRNA and lncRNA gene expression. The gene IDs were transformed to gene names based on the information in the ensemble database (http://asia.ensembl.org/info/data/index.html). Based on the m6A-related gene type of ‘writers’, ‘readers’, and ‘erasers’ and the corresponding gene name, m6A-related gene expression data were extracted with the limma package in R software (https://www.r-project.org/). A co-expression analysis was conducted to identify the correlation of m6A-related gene expression with lncRNAs. With the correlation coefficient, we derived the regulatory hypotaxis between the two factors. By this method, we acquired the expression data of m6A-related lncRNA through the limma package in R software (http://bioconductor.riken.jp/packages/3.0/bioc/html/limma.html). Moreover, the network plot was depicted via the igrah package to visualize the correlation. Expression data of m6A-related lncRNA were combined with the clinical survival data via the limma package. Prognosis-related lncRNAs were extracted, and the confidence interval and hazard ratio were calculated by using the survival package. The differences in the expression of m6A-related prognostic lncRNAs between the tumor and the normal tissue specimens were ascertained through the limma package, pheatmap package, reshape2 package, and ggpubr package in R software. Differences with *p* < 0.05 were considered to be statistically significant. to intuitively comprehend the difference, we drafted a heatmap and a boxplot.

### Role of m6A-related lncRNAs

Initially, we sorted the prognosis-related m6A-lncRNAs into two subtypes, cluster 1 and cluster 2, via the ConsensusClusterPlus and limma package according to the expression of the lncRNAs (https://bioconductor.org/install/) using the clusterAlg = ‘km’ and clusterNum = ‘2’. We executed the survival analysis according to the subtypes of lncRNAs via survminer, and used a survival package to evaluate the prognostic value of m6A-related lncRNAs. To identify differences in the expression of prognosis-related lncRNAs in each cluster and to analyze the relationship between lncRNAs and clinicopathological parameters, pheatmap package was utilized and a heatmap was created. Differences in the expression of target genes of the related subtype and different types of tissue were identified via the limma package. The standard name of the target gene was obtained from the NCBI (https://www.ncbi.nlm.nih.gov/) database. In order to clarify the correlation between target gene and prognostic m6A-related lncRNAs in HCC, a gene correlation analysis was conducted using the limma package. Differences were statistically significant when the *p*-value was less than 0.05.

### Role of immune cell infiltration and the TME

To explore and calculate the infiltration of different immune cell in the tissue samples, we used preprocessCore, limma, and the e1071 package and found that there was some amount of immune cell infiltration. The analysis of TME was carried out through ESTIMATE and limma package, and the stromalscore, immunescore, and ESTIMATEscore were obtained. The abovementioned scores were inversely related to tumor purity. The differences in immune cell infiltration of different clusters were analyzed using limma package and are depicted using vioplot. Simultaneously, the infiltration of each type of immune cell in different clusters of HCC was analyzed via limma package and is depicted as a boxplot. Thereafter, we conducted an analysis of the differences in the TME in different subtypes of the samples using limma package based on the immune score, ESTIMATE score, and stroma score, and corresponding boxplots were achieved to further explore the purity of tumor cells in the different TME subtypes. Gene set enrichment analysis (GSEA) (https://www.gsea-msigdb.org/gsea/index.jsp) was conducted to identify the differences of related functions and pathways in different samples, and data were imported via PERL software. The related score and plots were obtained to evaluate whether functions and pathways were dynamic in different clusters (c2.cp.kegg.v.7.2.symbols.gmt, cluster.cls#C2 versus C1). Each sample was defined as either ‘H’ or ‘L,’ depending on whether it was a high-risk cluster of prognosis-related lncRNAs. The number and type of permutations was set at ‘1000,’ ‘no collapse,’ and ‘phenotype,’ respectively. The gene list order was in the ‘descending’ mode and the gene list was sorted in ‘real’ mode. The metric for ranking the genes was ‘Signal2Noise.’ The normalization mode was ‘meandiv,’ and an FDR <0.05 indicated that the difference was statistically significant.

### Development of the m6A-lncRNA-related prognostic model

Lasso regression was undertaken to construct a prognostic model. All of the samples were divided into either a high-risk group or a low-risk group according to the median value of the risk score of the prognostic m6A-lncRNAs. Training (50%) and test (50%) groups were defined in Lasso regression, and the related plots were obtained. The survival curves of the high-risk and low-risk groups were obtained and compared. In order to evaluate the accuracy of our model to predict the survival in HCC, a corresponding receiver-operating characteristics (ROC) curve was obtained via the timeROC package. A risk curve was obtained based on the risk score, and the survival status and risk associated with the m6A-lncRNAs were evaluated with regard to the curve. An independent prognostic analysis was conducted to evaluate whether our model was independent of other clinical prognostic factors that could potentially affect the patients’ outcome. Multivariate and univariate analyses were conducted, and hazard ratios were calculated. Model validation for clinical groups was utilized to test and verify whether our model could be applied to different clinical groups. Risk and clinical correlation analyses were carried out to distinguish between the related high-risk and low-risk m6A-lncRNAs and to expound the correlation of the clinical characteristics and our prognostic risk model. Heatmap was depicted via the pheatmap package and the limma package. Boxplots of risk and clinical relevance were obtained to evaluate the risk based on the clinical data. Genetic difference analysis was obtained to assess the differences in target gene expression in different risk groups of our model in HCC patients. Correlation analysis of the risk and immune cells was conducted to evaluate the relationship between the immune cells and the risk score, and was presented as a scatterplot.

## RESULTS

In this study, we aimed to illustrate the influence of m6A-lncRNAs and immune cell infiltration in HCC. We identified 22 m6A-related prognostic lncRNAs and 10 high-risk m6A-lncRNAs, based on the differences in their expression between tumor tissues and normal tissues. A related prognostic model was developed via lasso regression, and GSEA was conducted to identify latent signaling pathways that might be involved in the development and progression of HCC. To explore and calculate the infiltration of different immune cells in the samples, the TME was analyzed.

### Identification of m6A-related lncRNAs

**Figure 1.**
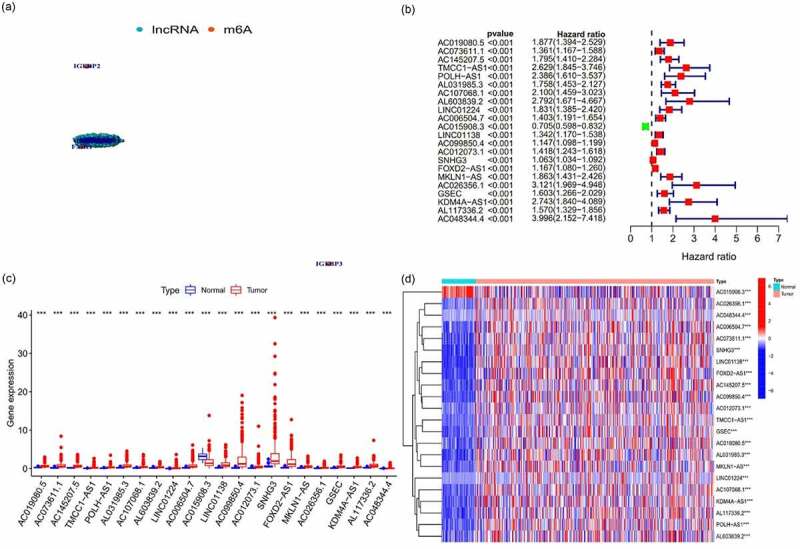
The expression of m6A-long noncoding RNAs (lncRNAs) and their role in the prognosis of hepatocellular carcinoma patients

The m6A-related gene expression data were extracted from the collated transcriptome data to distinguish between mRNA and lncRNA. A network plot was drawn to identify the correlation among m6A-related gene expression and lncRNAs (Figure 1a). The results of univariate Cox regression analysis are shown in a forest plot (Figure 1b). The lncRNAs were regarded as m6A-related prognostic lncRNAs when *p* < 0.05. The differences between tumor and normal tissues in the expression of the m6A-related prognostic lncRNAs were identified and are shown as heatmap and boxplot (Figures 1c and 1d). There were 22 m6A-related prognostic lncRNAs, and their expression differed in tumoral and normal tissues. Some lncRNAs were highly expressed in the tumor whereas others were highly expressed in normal tissues (*p* < 0.05).

**(A)**: Network plot of correlation among m6A-related gene expression and lncRNAs. **(B)**: Forest plot of univariate Cox regression analysis. Data on prognostic lncRNAs were extracted, and the confidence intervals and hazard ratios were calculated. Red represents high risk, whereas green represents low risk. **(C)**: Boxplot of the differences in the expression of m6A-prognostic lncRNAs in the tumor and normal tissues. *p < 0.05; **p < 0.01; ***p < 0.001. **(D)**: Heatmap of the differences in the expression of m6A prognostic lncRNAs between tumor and normal tissues. *p < 0.05; **p < 0.01; ***p < 0.001. Red represents high expression, whereas blue represents low expression. The abscissa represents the sample, whereas the ordinate represents prognostic lncRNAs.

### Role of m6A-lncRNAs

**Figure 2.**
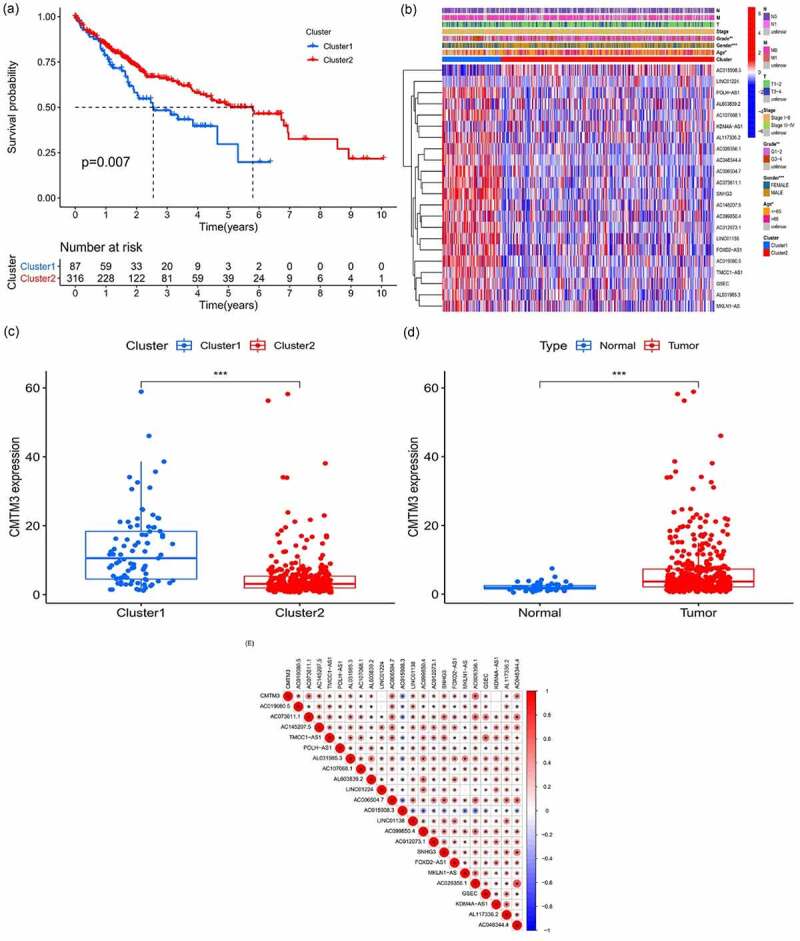
The expression and relationship of m6A prognostic long noncoding RNAs (lncRNAs) and target gene

According to the expression of lncRNAs, when K = 2, there was least overlap between the two types, and the CDF value was lowest; therefore, we classified lncRNAs into two types: cluster 1 and cluster 2. Survival analysis according to the lncRNA subtypes was undertaken to evaluate the prognostic value of m6A-lncRNAs, and the survival rate of cluster 1 was higher than that of cluster 2 (*p* = 0.007), as shown in Figure 2a. Heatmap depicts the differences that were identified in the expression of prognosis-related lncRNAs and the relationship was analyzed with regard to clinicopathological parameters (Figure 2b). Some lncRNAs were highly expressed in cluster 1 whereas others were highly expressed in cluster 2. In Figure 2b, there was no difference in the expression of prognosis-related lncRNAs in the different clusters, although these lncRNAs were related to age, sex and grade (*p* < 0.05). Differences in the expression of target genes in the related subtypes and in different types of tissue specimens are shown in Figures 2c and 2d. CMTM3 expression was lower in normal tissue than in the tumor (*p* < 0.001), whereas the expression of the abovementioned gene was higher in cluster 1 (*p* < 0.001). Gene correlation analysis was conducted to ascertain the correlation between the target gene and the prognostic m6A-lncRNAs in HCC (Figure 2e), and we found that the abovementioned target gene is related to prognosis-related m6A-lncRNAs (*p* < 0.05).

**(A)**: Survival analysis according to subtypes of lncRNAs, the survival rate of cluster 2 was higher than that of cluster 1, *p* = 0.007. **(B)**: Heatmap of differences in the expression of prognostic lncRNAs and the relationship with clinicopathological parameters in different clusters. *p < 0.05. Red represents high expression, whereas blue represents low expression. The abscissa represents the sample, whereas the ordinate represents prognostic lncRNAs. **(C + D)**: Differences in the expression of the target gene in related subtypes and different types of tissues. The expression of *CMTM3* in normal tissue was lower than that in tumoral tissues (*p* < 0.001), whereas the expression of the abovementioned gene was higher in cluster 1 (*p* < 0.001), which means that *CMTM3* might be an oncogene for hepatocellular carcinoma. **(E)**: Correlation analysis to analyze the correlation between target gene *CMTM3* and prognostic m6A-lncRNAs in hepatocellular carcinoma. Red means a positive correlation, whereas blue means a negative correlation, *indicates a statistically significant difference.

### Role of immune cell infiltration and TME

**Figure 3.**
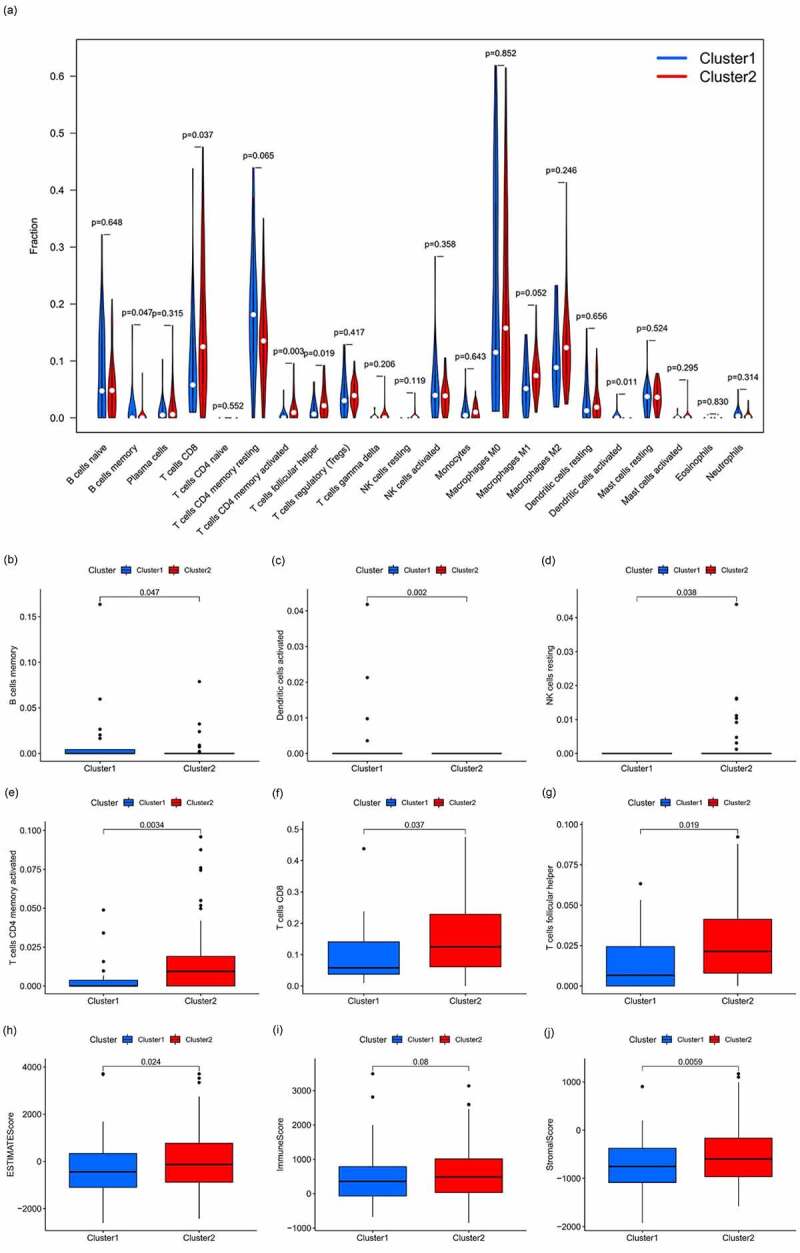
Analysis of immune cell infiltration and the tumor microenvironment

**Figure 4.**
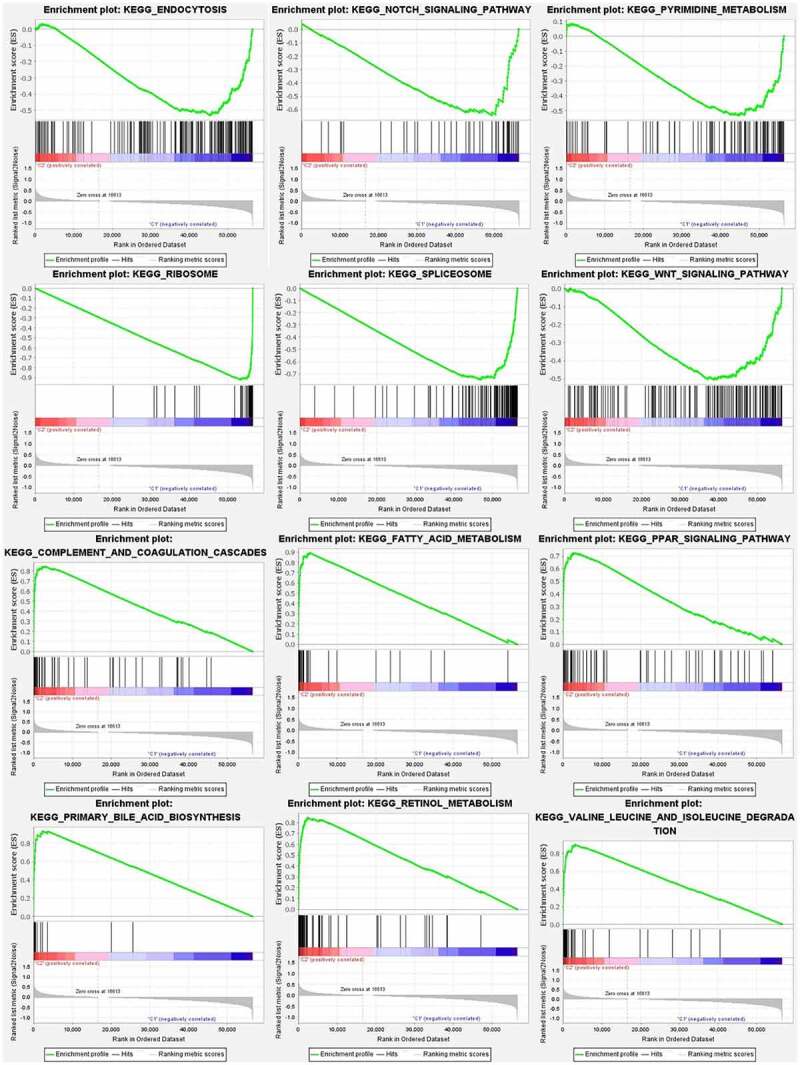
Gene set enrichment analysis

The differences in immune cell infiltration in different clusters were analyzed and a vioplot was obtained (Figure 3a). The infiltration of each type of immune cell in different clusters of HCC was analyzed and depicted as a boxplot (Figure 3b–g); immune cells, such as memory B cells, resting NK cells, and activated dendritic cells were highly clustered in cluster 1 (*p* < 0.05), whereas activated CD4 memory T cells, T cells CD8, and follicular helper T cells were highly clustered in cluster 2 (*p* < 0.05); there was no intergroup difference for the other cells that were analyzed (*p* > 0.05). We conducted analyses of the difference in the TME in different sample subtypes, and corresponding boxplots were drawn to further explore the purity of tumor cells in different specimen types (Figure 3h–j). All of the scores are higher in cluster 2, which means a lower purity of tumor cell and more immune-related cells in the TME (*p* < 0.05). Then, GSEA was conducted to clarify the differences in the related function and pathways in different samples (Figure 4). The top 6 enriched functions or pathways of each cluster are listed. Both FDR q-value and FWER *p*-value were <0.05. In the result, the most enriched pathway was the ‘NOTCH SIGNALING PATHWAY,’ and some of them were positively related to C1 or C2.

**(A)**: Vioplot of the analysis of differences in immune cell infiltration in different clusters. **(B–G)**: Boxplot of the analysis of differences in immune cell infiltration in different clusters. Memory B cell, resting NK cells, and activated dendritic cells highly infiltrated cluster 1, whereas activated memory CD4 T cells, CD8 T cells, and follicular helper T cells highly infiltrated cluster 2, *p* < 0.05. **(H-J)**: Analysis of differences in the tumor microenvironment in different subtypes. The ESTIMATE score and Stroma score were higher in cluster 2, which indicates a lower purity of tumor cells (*p* < 0.05).

To clarify the difference of related function or pathway in different samples, the top 6 enriched functions or pathways of each cluster were listed. The most enriched pathway was the Notch signaling pathway. Both FDR q-value and FWER *p*-value were <0.05.

### m6A-lncRNA-related prognostic model

**Figure 5.**
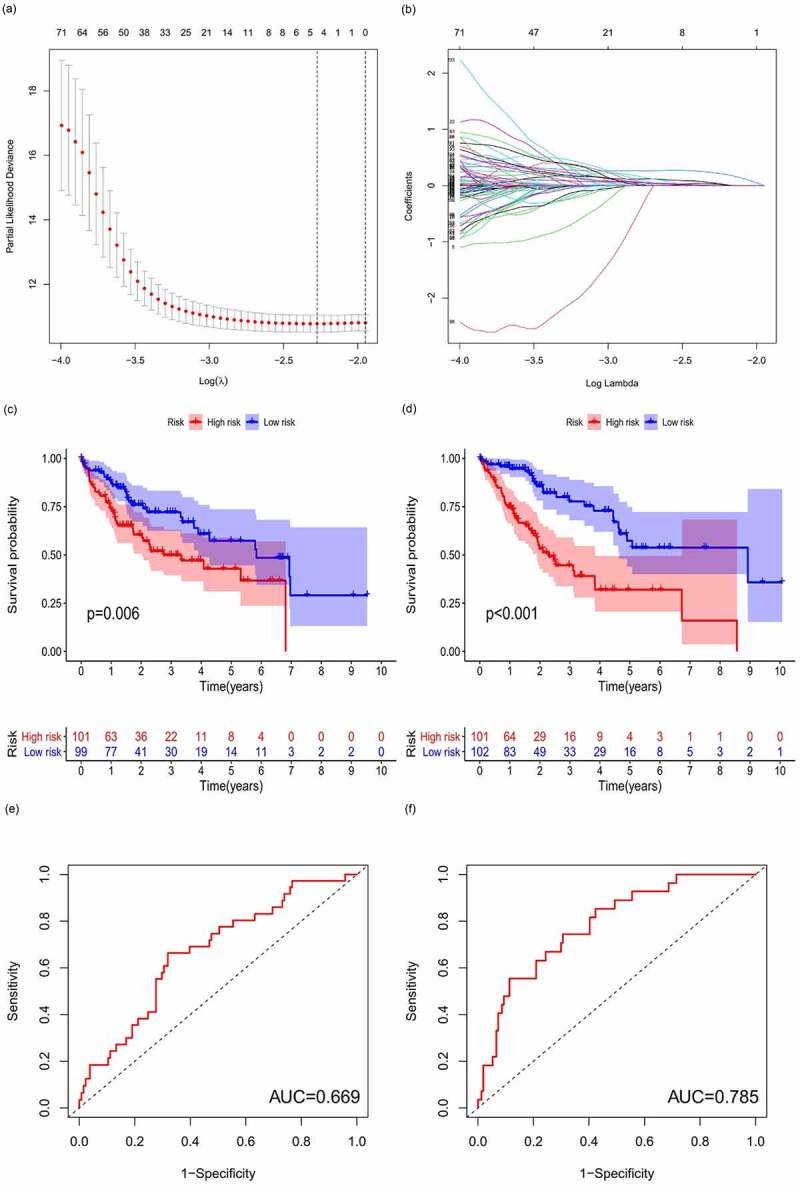
Prognostic model and its influence on the prognosis of HCC patients

**Figure 6.**
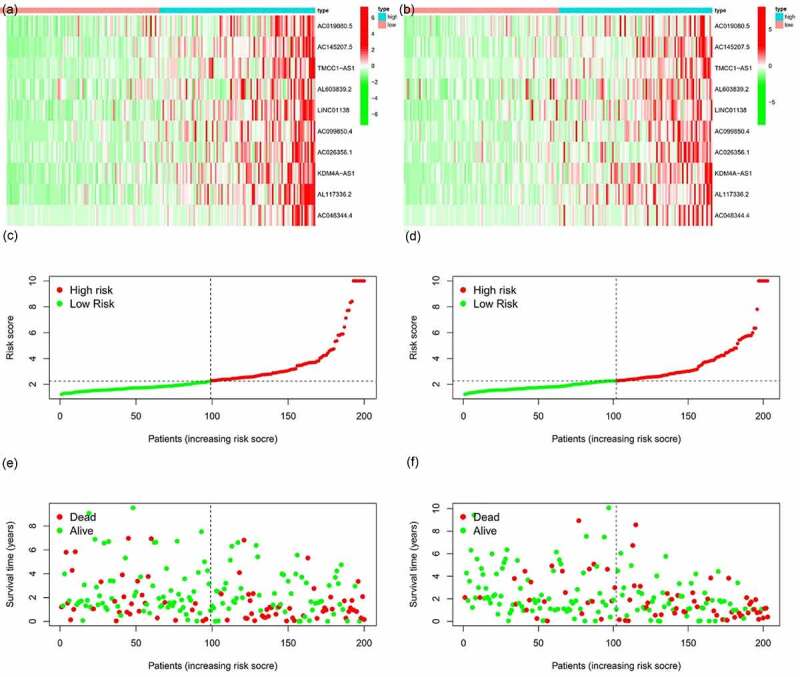
m6A-lncRNA-related risk score and its influence on the prognosis of HCC patients

**Figure 7.**
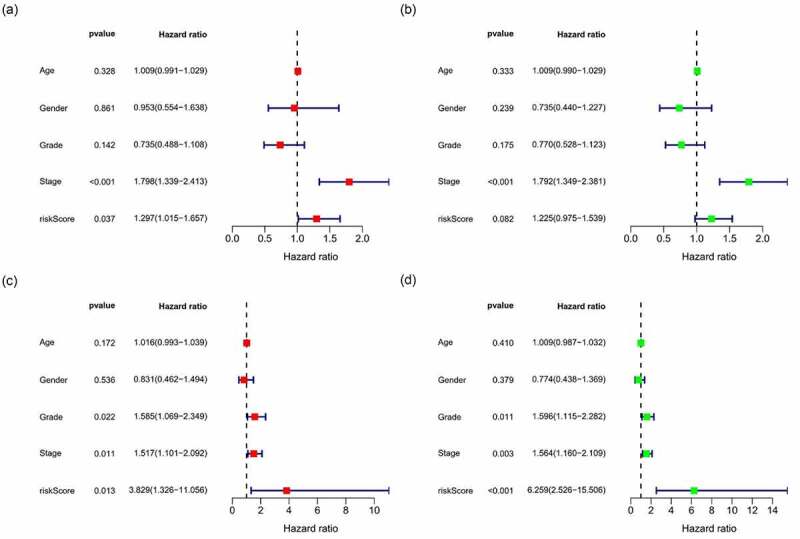
Multivariate and univariate analyses of independent prognostic analysis

**Figure 8.**
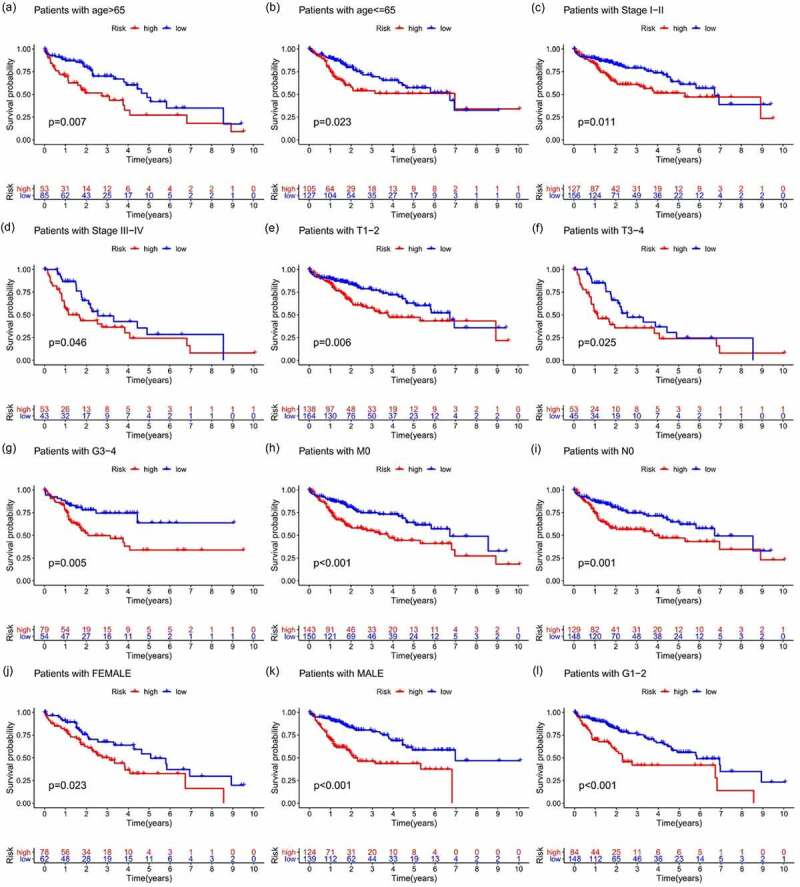
Survival curve for model validation

**Figure 9.**
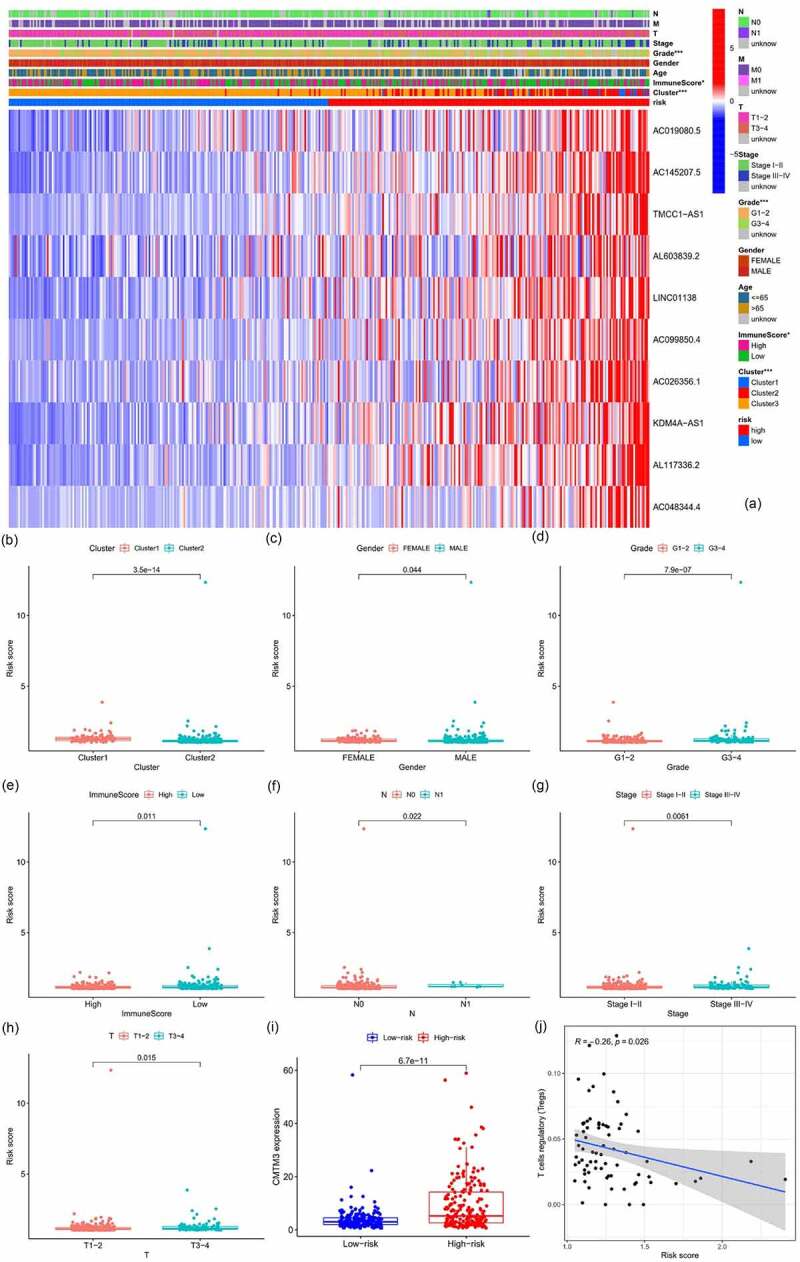
Correlation analysis of risk, immune cells, and clinical and genetic differences analysis of the target gene

All samples were divided into either the high-risk group or the low-risk group according to the median value of the risk score of prognostic m6A-lncRNAs via lasso regression. There were training (50%) and test (50%) groups in lasso regression, and related plots were obtained (Figures 5a and 5b). The survival curves were compared for the high-risk and low-risk groups (Figures 5c and 5d). Both in the test group and the training group, the survival rate of the low-risk subtype was higher than that of the high-risk subtype (*p* < 0.05). In order to evaluate the accuracy of our model to predict the survival of patients with the disease, a corresponding ROC curve was obtained via timeROC package (Figures 5e and 5f), wherein both the areas under the curve (AUCs) were >0.5 and attest to the considerable accuracy of our model in predicting survival with the disease. The risk curve was achieved, and the survival status and risk of m6A-lncRNAs were assessed based on the curve in Figure 6. As the risk score increases, the number of deaths increases and the ratio of high risk increases. The m6A prognosis-related lncRNAs were high risk. Independent prognostic analysis was conducted to evaluate whether our model was independent of other clinical prognostic factors that could affect the patients’ outcome (Figure 7). Stage, grade, and risk score are independent prognostic risk factors for HCC (*p* < 0.05). Model validation for clinical groups was utilized to test and verify whether our model could be applied to different clinical groups (Figure 8), and we confirmed that our model could be applied to different clinical groups stratified by: age, sex, lymph node metastasis, stage, grade, and T stage (*p* < 0.05). Risk and clinical correlation analyses were conducted to clarify the related high-risk and low-risk m6A-lncRNAs and to expound the correlation of clinical characteristics and our prognostic risk model (Figure 9a–h). Genetic difference analysis was conducted to assess the difference in the expression of target genes in different risk groups of our model in HCC (Figure 9i). The CMTM3 expression was higher in the high-risk group (*p* = 6.7e-11); the scatterplot depicts this association and facilitates an assessment of whether immune cells are beneficial or detrimental (Figure 9j). Immune cells such as regulatory T cells (Tregs) were negatively associated with the risk score (R = −0.26 and *p* = 0.026).

**(A + B)**: A prognostic model was constructed via lasso regression. **(C + D)**: Survival curve of different groups. In both groups, the survival rate of low-risk patients was higher than that of high-risk patients (**C**: test group, **D**: training group, *p* < 0.05). **(E + F)**: Receiver operating characteristics curve to evaluate the accuracy of our model in predicting the survival of patients with the disease. (**E**: test group, **F**: training group, area under the curve >0.5). This model can accurately predict the prognosis of HCC patients.

(**+ D**) Risk-related heatmap: m6A-lncRNAs, such as AC019080.5, AC145207.5, TMCC1-AS1, AL603839.2, LINC01138, AC099850.4, AL117336.2, AC026356.1, AC048344.4, and KDM4A-AS1, were highly expressed in the high-risk group, which means all of them might be detrimental to prognosis of HCC patients. **(B + E)** Risk-related curve and **(C + F)** risk-related spot plot. With an increase in the risk score, the number of deaths increases and the ratio of high risk increases. **A + B + C**: test group, **D + E + F**: training group.

(**A + B)**: in the test group, both stage and risk score were risk factors for the prognosis of HCC patients; **(C + D)**: in the training group, grade, stage and risk score were risk factors, *p* < 0.05; **(A + C)**: multivariate analysis, **(B + D)**: univariate analysis.

Our model could be applied to different clinical groups: age **(A + B)**, grade **(G + L)**, lymphatic metastasis **(I)**, M stage **(H**), stage **(C + D**), sex **(J + K**), and T stage **(E + F)**, in different groups by age, sex, grade, lymph node metastasis, M stage, and T stage; the survival rate of the low-risk group was higher than that of the high-risk group, *p* < 0.05.

**A**: Heatmap of risk and clinical correlation analysis, where the risk score was closely related to grade, cluster, and immunescore, *p* < 0.05(*p < 0.05, ***p < 0.001); **B-H**: Boxplot of risk and clinical correlation analysis. The risk score was closely related to grade, sex, T stage, stage, N stage, immunescore, and cluster; *p* < 0.05. **I**: Analysis of genetic differences in target gene. The expression of CMTM3 was higher in the high-risk group (*p* = 6.7e-11). **J**: Scatterplot of correlation analysis of risk score and immune cells. Regulatory T cells are negatively related to the risk score, R = −0.26 and *p* = 0.026.

## DISCUSSION

The high incidence and mortality of HCC seriously endangers human health. Surgery is considered to be the only radical cure for HCC, but is associated with complications, such as pancreatic fistula, biliary fistula, intestinal obstruction, early tumor recurrence, and other serious postoperative complications, which seriously reduced the survival rate and affected the prognosis of HCC patients. After tumor downstaging, liver transplantation, compared with non-transplantation therapies, improved the survival of HCC patients for the post-downstaging carcinoma response and could be dedicated to the extension of the HCC transplantation criteria [11]. m6A modification is accurately regulated by copious ‘writers,’ ‘erasers,’ and ‘readers,’ which are involved in various steps of mRNA metabolism. Additionally, m6A modification influences the processing of lncRNA. m6A dominates cellular proliferation and maturation, both of which are linked with cancer development. Consequently, the regulation of m6A modification in cancer cells would have a profound impact on the progress of research into malignant tumors [12]. A growing number of studies have announced the pathological significance of m6A in cancers including HCC, and m6A-related genes could be prognostic markers for predicting survival [13]. LncRNA is one of the major classes of non-coding RNAs and plays vital roles in chromatin organization as well as transcriptional and posttranscriptional regulation [14]. Earlier studies mainly focused on the correlation between specific m6A-related genes or pathways as well as tumor diagnosis and treatment. Thus, the lack of evidence from a systematic analysis of m6A-related lncRNAs in HCC should be solved imminently. The recognition and analysis of m6A-related lncRNAs in large HCC cohorts are of considerable significance in guiding the direction and targets for HCC research.

In this study, we extracted m6A-related gene expression data and distinguished between mRNA and lncRNA. Co-expression analysis was conducted to identify the correlation between m6A-related gene expression and lncRNAs. In the co-expression network plot, we found an interesting phenomenon whereby various lncRNAs were linked with m6A-related genes in HCC. Therefore, this stimulated our marked research interest in the expression of m6A-related lncRNA and related functions in HCC. Data on prognosis-related lncRNAs were extracted and the confidence interval and hazard ratio were calculated. Univariate Cox regression analysis indicated that m6A-related lncRNAs were closely related to the prognosis of HCC. Cancers have many essential links with m6A modifications. m6A is considered to influence lncRNA splicing, which might alter cancer progression [15,16]. This study revealed 22 prognosis-related m6A-lncRNAs, whose expression differed between tumor tissues and normal tissues. Some lncRNAs were highly expressed in the tumor, whereas others were highly expressed in normal tissue (*p* < 0.05). Hypoxia-inducible factors induced YTHDF1 expression correlated with hypoxia-induced autophagy and related HCC progression by stimulating the translation of the autophagy-related genes ATG2A and ATG14, which are m6A-modified genes [17]. Recently, it was identified that triclosan exposure induces lipid-metabolism disorder via downregulation of miR-30b expression to regulate FTO-mediated m6A methylation, which might be a feasible molecular mechanism in HCC [18]. Furthermore, m6A modification influences liver carcinoma and lipid metabolism in hepatic disease as well as the potential therapeutic strategies. As a vital component of the m6A methyltransferase complex, circular RNAs (circRNAs) has been demonstrated recently to stimulate HCC progression [19]. m6A regulates HCC metastasis through a related signaling pathway and promotes incursive phenotypes by triggering epithelial–mesenchymal transition, which mediates the differentiation of HCC and drug resistance [20]. The m6A modification of lncRNAs plays a vital role in altering the lncRNA structure and affects their interaction with proteins by mediating the repression of gene transcription and altering its subcellular distribution [21]. The abovementioned studies well explain the results of our study in that some m6A-lncRNAs are overexpressed in tumors whereas others are highly expressed in normal tissue. In addition, m6A-lncRNAs may act as oncogenes or tumor suppressors.

We further explored the role of m6A-lncRNAs in HCC. Survival analysis according to the lncRNA subtypes was conducted to evaluate the prognostic value of m6A-lncRNAs. Low-risk lncRNAs are beneficial to the prognosis of HCC. Besides, lncRNAs are closely related to the patient outcome in HCC. The results of our study are consistent with those reported by Liu et al. in that an m6A-related gene inhibits matrix metalloproteinase mediated hepatocellular carcinoma progression on combination with Human antigen R [19]. Additionally, Zhou et al. [20] indicated that m6A RNA methylation-mediated HNF3γ decreases HCC dedifferentiation and sorafenib resistance. There was no difference in the expression of m6A-lncRNAs in different clusters, possibly because most of the m6A-lncRNAs were poorly expressed in HCC in our study. There are only a few studies of m6A-related modification of lncRNAs. Thus, there is an urgent need for further research required for exploring the exact mechanism of m6A-related lncRNA modification and recognition to validate our results.

CMTM3 is a member of the CMTM family that is located at 16q22.1. CMTM3 strongly curbed the colony-forming ability of cancer cells, and this inhibited tumor cell proliferation and mediated apoptosis with caspase-3 activation via promotion of CpG methylation in cancer [22]. CMTM3 could be advantageous for successfully developing drug candidates into therapeutic anticancer strategies. In this study, CMTM3 expression was higher in the HCC tumor sample and in cluster 1, which means that CMTM3 might be an oncogene. However, CMTM3 expression was higher in the high-risk group, and the fact that CMTM3 is highly expressed in various HCC cells might explain this condition. From the outcome of the correlation analysis of a conjunction between the target gene and prognostic m6A-lncRNAs in HCC, CMTM3 was seen to be associated closely with the abundant m6A-lncRNAs. With the increased expression of abovementioned positively related m6A-lncRNAs in HCC cells, the expression of CMTM3 also increased. The results of m6A-lncRNAs-related risk score indicated that m6A-lncRNAs, such as AC019080.5, AC145207.5, TMCC1-AS1, AL603839.2, LINC01138, AC099850.4, AL117336.2, AC026356.1, and AC048344.4, were highly expressed in the high-risk group and, thus, all of them might be oncogenes for HCC. This finding further supports our hypothesis that CMTM3 may be an oncogene. Additionally, the abovementioned m6A-lncRNAs might be a therapeutic target for HCC. CMTM3 could play an essential role in the migration and invasion of HCC cells by repressing the EMT phenotype via suppression of the JAK2/STAT3 signaling pathway, which induces the overexpression of the E-cadherin and decreases the expression of the mesenchymal marker N-cadherin [23]. However, few studies have evaluated CMTM3 and, therefore, the specific molecular mechanisms underlying the abovementioned function that leads to tumor suppression needs to be clarified in further research studies.

Furthermore, we explored and calculated the infiltration of different immune cells in the samples to identify the role of immune cell infiltration and the TME in HCC. Analysis of the differences in immune cell infiltration indicated that immune cells, such as memory B cells, resting NK cells, and activated dendritic cells highly infiltrated the tumor tissues in cluster 1, whereas activated CD4 memory T cells, CD8 T cells, and follicular helper T cells highly infiltrated samples in cluster 2. Therefore, the infiltration of activated CD4 memory T cells, CD8 T cells, and follicular helper T cells in the TME may be detrimental to the prognosis of HCC patients. Our inference is in line with the conclusion of Liu et al. that intratumoural immunostimulated dendritic cells were critical for effective tumor rehabilitation, and improved immunotherapy with activated dendritic cells obtained a considerably stronger immune response against HCC cells [24]. Moreover, Zhang et al. opined that high densities of tumor-infiltrating memory B cells signified an improved clinical outcome, which was associated with exceptional survival, and the high density of B cells correlated with a smaller tumor dimension and well-differentiated tumor that was beneficial for therapeutic strategies to target B cells in HCC [25]. Follicular helper T cells were highly clustered in the high-risk HCC group, which might provide a latent prognostic marker and constitutes a positive development for HCC patients [26]. An analysis of the differences in the TME in different HCC subtypes was conducted to further explore the purity of tumor cells in different HCC types. The ESTIMATE score and Stroma score were higher in cluster 2, indicating a lower purity of tumor cells and greater infiltration of immune-related cells in the TME of cluster 2. The intermediate stromal score and immune score were independent risk factors for disease-free survival and overall survival of HCC patients that displayed remarkable discriminatory power, accuracy, and clinical effectiveness for predicting efficacy of sorafenib treatment and molecular alterations of hepatocellular carcinoma [27]. The results of the abovementioned studies support our hypothesis that immune cell infiltration of the TME influences the prognosis of HCC patients. The higher the immune score, the lower the purity of the tumor and, eventually, the better the prognosis.

In GSEA, the Notch-signaling pathway was found to be the most significantly enriched pathway and was an essential molecular pathway of embryonic development in humans, and the overexpression of Notch in HCC cells results in increased cell proliferation [28]. The Notch signaling pathway regulates cancer biology, including aspects such as cancer progression and metastasis, and influences the TME, which might provide an essential therapeutic target [29]. There are four Notch receptors and various ligands and some of them participate in HCC outcome based on their regulation by a related upstream gene and significantly suppress cell proliferation, invasion, and accelerate HCC progression [29]. Taking the abovementioned factors into account, m6A-lncRNAs may function by regulating the Notch signaling pathway to influence the migration and proliferation of HCC cells. The m6A-lncRNA-related prognostic model was developed via lasso regression. Both in the test and training groups, the survival rate of the low-risk subtype was higher than that of the high-risk subtype. The m6A-lncRNA-related prognostic model can predict the outcome of HCC. Additionally, the accuracy of our model for predicting survival of HCC patients is considerable. An increase in the risk score is associated with an increase in the number of deaths and the ratio of high risk. Going a step further, our model was independent of other clinical prognostic factors that can affect patient outcomes. Therefore, the model could be applied to different clinical groups. The m6A modification of lncRNAs may alter lncRNA structure and affect their interaction with proteins, which may mediate the repression of gene transcription [30,31]. The m6A modification of lncRNAs possibly alters their subcellular dissemination, which regulates lncRNA stability and promotes tumorigenesis and metastasis [32]. In summary, the evidence in the literature and the results of our research showed that m6A-lncRNAs could be a suitable clinical model to predict the outcome of HCC patients. The result of an analysis of genetic differences indicated that the expression of the CMTM3 gene is higher in the high-risk groups of our model in HCC. This result contradicts the conclusion of Li [23] that CMTM3 inhibits the proliferation and tumorigenesis of HCC. This is possibly because CMTM3 was highly expressed in the HCC samples in our study. Considering the paucity of researches on the CMTM3 gene and because no related study has explored the role of CMTM3 in HCC cells, the results of this study form the basis of a new research direction. Correlation analysis of risk and immune cells was conducted to evaluate the relationship between immune cells and risk scores. Regulatory T cells are negatively related to the risk score. This is consistent with the result of the analysis of differences in immune cell infiltration in different clusters. Regulatory T cells upregulate checkpoint inhibitors via several inhibitory pathways and are conducive to systemic immune dysfunction and the deterioration of antitumoral activity that, in all probability, accelerates HCC progression and a poor prognosis [32]. Thus, selective strategies to block or knockdown related genes or signaling pathways that interfere with regulatory T cells in HCC patients to ameliorate antitumoral activity and possibly jeopardize immunosuppression in the TME.

## CONCLUSION

We investigated prognosis-related m6A-lncRNAs by analyzing the expression profiles and clinical data of HCC samples from the TCGA database. Immune cell infiltration was studied, and a prognostic model was developed to confirm the role of m6A-lncRNAs in HCC. Moreover, we showed that CMTM3 is highly related to overall survival. The analysis of differences in expression as well as correlation analysis further clarified the function of prognostic CMTM3. Our target gene *CMTM3* was positively associated with the risk-related m6A-lncRNAs AC019080.5, AC145207.5, TMCC1-AS1, AL603839.2, LINC01138, AC099850.4, AL117336.2, AC026356.1, and AC048344.4, whereas it was negatively associated with AC015908.3. Our study strengthens the understanding of m6A-related lncRNAs and immune cell infiltration in the TME, which might provide novel therapeutic targets and prognosis-related biomarkers for evaluation in further research.

### Consent

The Cancer Genome Atlas is a public database and patients included in the database have provided informed consent for the use of their data. Users can freely download the relevant data for research purposes and publish relevant articles. As our study is based on open-source data, no ethical issues or conflicts of interest are reported.

## Supplementary Material

Supplemental Material

## Data Availability

The dataset supporting the conclusions of this article is available upon reasonable request from The Cancer Genome Atlas. https://portal.gdc.cancer.gov/

## Highlights

m6A-lncRNAs are closely related to HCC prognosis

The prognostic model showed m6A-lncRNAs are independent prognostic factors

Immune cell infiltration, target genes, and signaling pathway are therapeutic targets
