# Supplementary figures and images for: Comprehensive analysis of N6-methyladenosine -related long non-coding RNAs and immune cell infiltration in hepatocellular carcinoma
Source: Bioengineered. 2021 May 6;12(1):1708–24. doi: 10.1080/21655979.2021.1923381 (PMC8806206; doi:10.1080/21655979.2021.1923381)

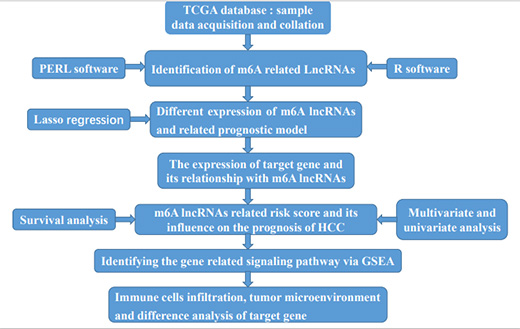

Supplement: Supplemental Material [file KBIE_A_1923381_SM0107.zip › Graphical Abstract.jpg]
