# Supplementary material for: Comprehensive analysis of N6-methyladenosine -related long non-coding RNAs and immune cell infiltration in hepatocellular carcinoma
Source: Bioengineered. 2021 May 6;12(1):1708–24. doi: 10.1080/21655979.2021.1923381 (PMC8806206; doi:10.1080/21655979.2021.1923381)
Supplement: Supplemental Material [file KBIE_A_1923381_SM0107.zip › supp.rtf]

Dear editors and reviewers,
Manuscript ID: KBIE-2021-0249
Manuscript title: Comprehensive analysis of N6-methyladenosine-related
long non-coding RNAs and immune cell infiltration in hepatocellular
carcinoma
Thank you very much for your kindness. Thank you for taking the
time to review our article from your busy schedule. It is our great honor
to submit the manuscript to your journal. Many thanks to the Referee for
encouraging our work and for giving useful comments for clarifying,
improving and correcting some materials in the paper. Thanks to all the
reviewers for their valuable comments, we have made changes in
accordance with the corresponding review comments. We have separated
our responses to the reviewers' comments according to several categories
in order to achieve an integrated approach in our responses. Now we
have carefully revised the paper according to your comments, as
explained below.
Reviewer 1:
Overall, the conclusions drawn by this paper are well-founded. m6A LncRNAs
are important regulators for progression of HCC. m6A LncRNAs and related
immune cell infiltration could provide potential therapeutic targets for cancer
treatments.
Re: We sincerely appreciate the reviewer's positive evaluation of our
work. We will spare no effort to do better.
While the effort of compiling this review is commendable and the topic
potentially interesting and relatively novel, I have some suggestions to help the
Supplementary Material - for review
authors to expend the content and improve the quality.
Re: Thank you very much for your suggestions to help us to expend the
content and improve the quality. We have checked the manuscript
carefully according to your advice and related revisions have been made,
please check them. If there are any other problems, please do not hesitate
to put forward.
1. Please ensure your references were correctly cited, if necessary, please add
more references necessary.
Re: Thank you very much for your suggestions. We apologize for the
mistakes in the part of references of the manuscript and also carefully
checked them. We have revised the references strictly in accordance with
the requirements of the magazine on the format of references.
2. Please improve English language usage.
Re: Thank you very much for your help. We apologize for the mistakes
in the manuscript and also carefully checked the entire manuscript for
typographic, grammatical and formatting errors. We apologize for the
language problems in the original manuscript. Following the reviewer's
suggestion, we have contacted the Taylor & Francis Editing Services
according to the advice of editors. Language presentation was improved
with assistance from Taylor & Francis Editing Services, whose unique
editor-selection algorithm ensures that our article was assigned to highly
qualified specialists with expertise in our subject and related outstanding
native English-speaking editors to deal with our manuscript.
Reviewer 2: Please provide comments for the author.
This study revealed the relationship between N6-methyladenosine lncRNAs and
immune cell infiltration in hepatocellular carcinoma. And the results showed
that m6A LncRNAs are closely related to occurrence and progression of HCC
and m6A LncRNAs and related immune cell infiltration in tumor
microenvironment can provide novel therapeutic target for further research.
There are several issues which need to be addressed. Listed below are major
points that really need to be answered or discussed followed by minor
comments.
Re: Thank you for your approval of our work and your valuable
comments. We have made the corresponding amendments in accordance
with your comments, please review.
Major modifications:
1. The purpose of this study is to analyze the relationship between m6A
lncRNAs and the occurrence and progression of HCC and to establish a
prognostic model related to m6A lncRNAs. Existing studies have confirmed that
related serum inflammation indicators (NLR, PLR, LMR, PNI, APRI, ANRI) and
pathological indicators (MVI) are related to the prognosis of HCC. We
recommended to compare the prognostic evaluation ability of the prognosis
model with the previously indicators.
Re: First of all, We would like to thank the reviewer for your precious
opinions and we need to thank the free download data service provided
by the TCGA database. It would be better to evaluate its prognostic
assessment ability via comparing the prognosis model in our study with
serum prognosis related inflammation indicators. Since our data comes
from the TCGA public database, the database does not provide data on
the above inflammation indicators, the above comparison cannot be
made for the time being. We can further confirm this in the follow-up
prospective research. In our next step research, the above indicators will
further be compared to evaluate each other's ability to judge the
prognosis.
2. This study evaluated and established a model of m6A lncRNAs related to the
survival status of HCC. It is recommended to use this model in patients with
HCC to verify the accuracy of this result.
Re: Thank you very much for your sincere comments. In this study, our
data comes from the TCGA database. We downloaded the corresponding
clinical data and transcriptome data. The clinical data includes clinical
data of patients diagnosed with liver cancer. Therefore, our research is
based on HCC patients. At the same time, we have also verified the
prognostic model in figure 8. Our model could be applied to different
clinical groups: age, grade, lymph node metastasis, M stage and T stage,
in different group of age, grade, lymph node metastasis, M stage and T
stage, the survival rate of low risk group was higher than that of high
risk group, P<0.05.
3. This article is purely biological information analysis. It is recommended to
supply trials to improve the scientific nature of this research.
Re: Sincerely thank you for your valuable comments. First of all, we
would like to thank the reviewer for taking the time to review our article.
Regarding this issue, it is true that our article is purely biological
information analysis, which needs to be combined with basic experiments
to make the argument more convincing. There are 22 m6A related
lncRNAs in our research. In order to ensure the scientific and rigorous
of the experiment, we should verify them one by one, so we promise to
strictly follow your opinions next time, and related verification analysis
to further confirm the current results in the follow-up research. We have
tried very hard to supplement experiments to further demonstrate the
scientific nature of our conclusions, but objective experimental
conditions do not allow it. Hope you can forgive us. We will supplement
relevant experiments and continue research if experimental conditions
permit in the future.
4. Special emphasis is needed on grammar and sentence structure.
Re: Thank you for your valuable comments. We have asked the
magazine's department responsible for article editing services to modify
and polish the article. The paper has been edited to ensure that the
language is clear and free of errors. The logical presentation of ideas
and the structure of the paper were also checked during the editing
process. The edit was performed by professional editors at Editage, a
division of Cactus Communications, in cooperation with Taylor &
Francis Group. The quality of the edit has been guaranteed. Please
review it. If there are any shortcomings, please raise them in time and we
will revise them as soon as possible.
Reviewer 3 (Editorials):
1- While submitting the revised manuscript, please double check the author
names provided in the submission so that authorship related changes are made
in the revision stage. If your manuscript is accepted, any authorship change
will involve approval from co-authors and respective editor handling the
submission and this may cause a significant delay in publishing your manuscript.
Re: Thank you very much for your notification. We have checked the
author names and did not make any changes.
2- Provide research highlights. The highlights should be 3 to 5 bullet points
maximum 85 characters, including spaces, per bullet point (practically in one
line). Highlights should reflect the main findings. They should not describe,
what was done. You may put the highlights in a separate word file or add to
the manuscript after the keywords. The highlights and graphical abstract
improve the visibility and citation of the work.
Re: Thank you very much for your valuable comments. The research
highlights have been added to the manuscript after the keywords
according to your advice. Please check it. Thank you once again.
3- Provide a graphical abstract. This is an image to give readers a clear idea of
the content of your article and it should be self explanatory. It should be a
maximum width of 525 pixels. If your image is narrower than 525 pixels, please
place it on a white background 525 pixels wide to ensure the dimensions are
maintained. Save the graphical abstract as a .jpg, .png, or .tiff. Please do not
embed it in the manuscript file but save it as a separate file, labelled Graphical
Abstract.
Re: Regardless of the result, we would like to thank you for your sincere
opinions. The graphical abstract with a maximum width of 525 pixels
has been saved as .jpg separately and sent to the journal with the revised
manuscript. Thank you very much.
4- Your manuscript needs a professional revision for the language. You may
use any professional service to improve its language. If you prefer to use
services offered by Bioengineered, you may refer to instruction for authors
or www.tandfeditingservices.com.
Re: Thank you very much for your recommendation. We have used
services offered by Bioengineered to make a professional revision for the
language. we have contacted the Taylor & Francis Editing Services to
help us to edit the manuscript. Our manuscript has been edited to ensure
that the language is clear and free of errors. The logical presentation of
ideas and the structure of the paper were also checked during the editing
process. Please check it.
5- Add clearly the hypothesis, aims and goals of this work to the last paragraph
to your introduction.
Re: Thank you sincerely for your hard work. We have added the above
information clearly as last paragraph of our introduction. Please check
it.
6- Check also recent similar publications in Bioengineered and compared your
work with them and clearly state your novelty compared to the previous works.
Re: Thank you very much for your guidance. By checking recent similar
publications in Bioengineered, we have summarized the following
novelties. We appraised prognosis related m6A lncRNAs by analyzing the
expression profiles and clinical data of HCC samples from TCGA
database. Immune cells infiltration and prognosis related model were
conducted to confirm the role of m6A related lncRNAs in HCC.
Functional and pathway GSEA further identified. Hereafter, we brought
to light that CMTM3 is highly related to overall survival. Difference
expression analysis and correlation analysis further clarified the
function of prognosis related CMTM3. Our study reinforces the
understanding of m6A related LncRNAs and immune cell infiltration in
tumor microenvironment, which might provide novel therapeutic target
and prognosis-related biomarker for further research.
7- Please add a starting paragraph to the results section to briefly introduce
the topic, your goals and hypothesis and a short summary of what you did in
this work. Many readers start reading the manus from the results and it should
be understandable.
Re: Thank you very much for your patience and guidance. It is true that
many readers start reading the manus from the results. In order to make
it more understandable, we have added a starting paragraph to the results
section to briefly introduce the topic, goals and hypothesis of our study.
Please check it. If there are any other problems, please don't hesitate to
enlighten us. We will spare no effort to work better to satisfy you.
8- The abbreviations in the title should also follow by full text (e.g.
Comprehensive analysis of N6-Methyladenosine (m6A) related long non-coding
RNAs (lncRNAs) and immune...)
Re: Thank you very much for your guidance. We have changed the title
according to your advice with the help of Taylor & Francis Editing
Services.
9- The abstract should be unstructured and all in one paragraph with max 250
words. Merge all the sections together.
Re: Thank you very much for your sincere advice. The abstract has been
unstructured and all in one paragraph with less than 250 words. Please
check it. Thank you very much for you hard work.
10- You have long paragraphs in your text that make it difficult for the readers.
Please cut them into shorter paragraphs.
Re: Thank you very much for your help. We are so sorry to make it
difficult for reading. We have short the paragraphs and words according
to the revise advice of edit service of Bioengineered. Please check it. Once
there are any other mistakes, please do not hesitate to inform us to correct.
It is very kind of you and your team to take time to deal with our
manuscript. We are very grateful for your hard work. We appreciate a lot
for your sincere advice and comments. We have tried our best to revise
the manuscript according to the comments of reviewers and yours. As a
proverb said, no one is perfect, no gold is pure. We look forward to
hearing from you regarding our submission. We would be glad to
respond to any further questions and comments that you may have.
Sincerely yours
First author: Zhong lin Yu
Corresponding author: Zheng ming Zhu
